# Transthyretin and Amyloid in the Islets of Langerhans in Type-2 Diabetes

**DOI:** 10.1155/2008/429274

**Published:** 2008-09-24

**Authors:** Gunilla T. Westermark, Per Westermark

**Affiliations:** ^1^Department of Clinical and Experimental Medicine, Linköping University, 581 85 Linköping, Sweden; ^2^Department of Genetics and Pathology, Uppsala University, 751 85 Uppsala, Sweden

## Abstract

Transthyretin (TTR) is a major amyloid fibril protein in certain systemic forms of amyloidosis. It is a plasma protein, mainly synthesized by the liver but expression occurs also at certain minor locations, including the endocrine cells in the islets of Langerhans. With the use of immunohistochemistry and in situ hybridization, we have studied the distribution of transthyretin-containing cells in islets of Langerhans in type-2 diabetic and nondiabetic individuals. TTR expression was particularly seen in alpha (glucagon) cells. Islets from type-2 diabetic patients had proportionally more transthyretin-reactive islet cells, including beta cells. A weak transthyretin immunoreaction in IAPP-derived amyloid occurred in some specimens. In seeding experiments in vitro, we found that TTR fibrils did not seed IAPP while IAPP fibrils seeded TTR. It is suggested that islet expression of transthyretin may be altered in type-2 diabetes.

## 1. INTRODUCTION

Deposition of amyloid is the single, most common, and characteristic morphological lesion in islets of Langerhans in individuals with type-2 diabetes. Some degree of islet amyloidosis is found in at least 95% of such patients [1]. In about two thirds of the cases, more than 50% of the islets are affected. As the amyloid amount increases, the percentage of beta cells decreases [2, 3]. The amount of amyloid can be considerable in a single islet, more or less converting it into amyloid.

The islet amyloid fibril consists of islet amyloid polypeptide (IAPP; amylin) which is a 37-amino acid residues beta cell hormone, stored together with insulin in secretory vesicles and released with this hormone. The normal molar ratio between IAPP and insulin in human is less than 5%. Although IAPP is a very fibrillogenic peptide in vitro, it does not fibrillize in normal islets, possibly due to interaction with insulin which is a potent inhibitor of IAPP fibril formation [4–6].

It is not understood why IAPP forms amyloid deposits in conjunction with type-2 diabetes. An overexpression of IAPP does exist in obese type-2 diabetic patients [7], but this alone is most probably not sufficient for amyloid formation. Thus, transgenic mice, highly overexpressing human IAPP, do not develop islet amyloidosis unless manipulated in other ways, for example, feeding a diet high in fat [8–10]. Therefore, additional factors may operate in the amyloidogenesis.

The amyloid diseases constitute a biochemically and clinically diverse group of disorders. Each amyloid disease is characterized by one specific amyloid fibril protein and until now, more than 25 proteins have been found in human amyloidosis [11]. Aggregation of a peptide to amyloid-like fibrils in vitro is a nucleation-dependent process in which a nucleus is formed before fibrils start to grow [12–14]. The nature of this nucleus is not fully understood and the time it takes for its formation, known as the lag phase, varies depending on protein, concentration, temperature, and other factors. As soon as a nucleus is present, the growth of amyloid-like fibrils can occur rapidly. Seeding a solution with preformed fibrils greatly enhances the fibrillogenesis and reduces the lag phase sometimes close to zero. It is assumed that protein monomers add to the free end of the fibrils. This mechanism is believed not only to work in vitro but also in vivo and may be an important reason why amyloid infiltration in systemic amyloidoses spreads rapidly as soon as it has started. Seeding capability is generally very specific and already minor variations in the protein efficiently block the addition of new monomers [15]. However, in vitro experiments have shown that preformed fibrils made from heterologous amyloid fibril proteins sometimes can act as seed [16]. Heterologous amyloid fibrils may also act as efficient seed in two different murine models of systemic amyloidosis [17, 18]. That means, at least in theory, that fibrils of one biochemical nature may be a risk factor for the formation of amyloid deposits of another kind of amyloidosis.

In addition to IAPP, another major amyloid fibril protein, transthyretin (TTR), is expressed in considerable amount in the pancreas [19, 20]. TTR is the major amyloid fibril protein in several systemic familial forms of amyloidosis and in the prevalent senile systemic amyloidosis [21]. Similar to other types of systemic amyloidosis, most of the fibril protein in these amyloidoses is derived from the plasma pool and the major expression site of TTR is the liver [22, 23]. There are, however, a few minor sites of TTR gene expression and one of them is the endocrine pancreas [24, 25]. The function of TTR in these localized cells is not known. Furthermore, the possible effect on amyloidogenesis by these additional expression sites has not yet been studied.

Against this background, we questioned whether IAPP-amyloid could induce TTR-fibril formation, and also whether deposition of TTR-amyloid in the pancreas leads to islet amyloidosis, either by deposition of fibrils from TTR or from IAPP. Since we were not aware of any study of islet TTR in type-2 diabetes, another aim was to study the immunoreactivity of islet alpha (glucagon) and beta (insulin) cells in diabetic and nondiabetic individuals.

## 2. MATERIAL AND METHODS

Paraffin-embedded pancreatic material (corpus or cauda) from 6 individuals with type-2 diabetes and from 10 nondiabetic individuals was available in the laboratory files. The inclusion criterion was that specimens had been taken within 12 hours after death. They were fixed in buffered neutral 4% formaldehyde solution and embedded in paraffin. Formalin-fixed and paraffin-embedded tissue from the pancreatic body was also obtained from two patients with long history of familial TTR-amyloidosis associated with a V30M-mutation in the TTR gene. Adjacent 5 *μ*m sections were taken for alkaline Congo red staining [26] and for immunohistochemistry. The study was approved by the Ethical Committee at Uppsala University Hospital.

Antibodies against insulin (guinea pig) and glucagon (rabbit) were purchased from DAKO (Glostrup, Denmark). Rabbit antisera (A110) against rat/mouse IAPP, which shows complete cross-reactivity with human IAPP [27] and against a recombinant C-terminal fragment (aa 50–127; antiserum # A1898) of human TTR, have been characterized previously [28]. A rabbit antiserum, A1899, was raised against a high-molecular fraction from a gel separation of ATTR fibrils from an individual with senile systemic amyloidosis. This antiserum recognizes TTR amyloid, but is unreactive with AL and AA amyloid in Western blot analysis. Immunohistochemistry was performed with the antisera diluted 1 : 2000 using the biotin-streptavidin system. Sections were developed with 3,3′-diaminobenzidine-tetrahydrochloride (DAB). Double immunolabeling was performed with antibodies against glucagon and TTR or insulin and TTR, visualized with rabbit, mouse, or Guinea pig secondary antibodies conjugated to Alexa 488 (analyzed in blue light) or Alexa 546 (analyzed in green light) (Molecular Probes, Eugene, Ore, USA).

For controls, sections were treated with antiserum preabsorbed with TTR. Cross-reactivity between TTR or IAPP and glucagon was ruled out by dot blot analysis for which TTR, IAPP, and glucagon were dissolved at 3 *μ* *g*/well in 0.1 M sodium carbonate buffer, pH 9.8, spotted on a nitrocellulose membrane and incubated with the antisera and developed with biotin-streptavidin followed by DAB. This showed that no cross-reactivity existed between IAPP and TTR or glucagon and TTR .

In order to test the specificity of the two TTR antisera, sections from an “amyloid array” were used. In this, 1 mm thick cylinders from amyloid-containing tissues had been embedded in one block. The array contained the following types of amyloid: AA-amyloid (7 cases, 9 tissues), AL-amyloid (7 cases, 9 tissues), ATTR-amyloid (6 cases, 3 tissues), A*β*-amyloid (1 case, brain), and IAPP-amyloid (3 cases, pancreas).

### 2.1. Degree of islet amyloidosis

The number of islets with and without amyloid deposits was determined in Congo red stained sections, analyzed in polarized light. Usually, at least 50 islets were scrutinized in each section.

### 2.2. Localization of TTR in islets of langerhans

A cDNA library was constructed from human liver with the aid of a cDNA synthesis kit (Amersham Bioscience, Uppsala, Sweden). A 237-nucleotide long fragment corresponding to amino acid residues 2–106 of TTR was amplified by polymerase chain reaction. The achieved fragment was ligated into the multiple cloning site of pGEM4Z (Promega, SDS-Biosciences, Falkenberg, Sweden). The vector was linearized in front of the SP6 or T7 promoters and digoxigenin-labelled RNA probes were produced according to the manufacturer (Roche, Bromma, Sweden). In situ hybridization was performed as described [29].

For ultrastructural studies, pancreatic tissue was available from one patient with type-2 diabetes and one normal control, fixed in 2% glutaraldehyde in phosphate buffer and embedded in epon. For double immunolabeling, ultrathin sections on formvar-coated nickel grids, were incubated with the primary antibodies (rabbit anti TTR50-127) and mouse antiglucagon (DAKO). TTR was visualized with 5 nm gold particles and glucagon with 10 nm gold particles (British Biocell, Cardiff, UK).

### 2.3. Seeding experiments

Full length human TTR was a kind gift from Dr. Tom Pettersson (Danderyd, Sweden) and human IAPP was synthesized by Keck Laboratory, New Haven, Connecticut . TTR (0.5 mg) and IAPP (0.125 mg) were dissolved separately in 25 *μ*l methyl sulfoxide (DMSO). To the solutions, 100 *μ*l distilled water was added. After incubation for 2 days at room temperature, 0.5 *μ*l samples were taken from each and studied electron microscopically (negative contrast) for presence of amyloid-like fibrils. Since typical fibrils were seen in both solutions, they were diluted to 2 ml with 0.05 M sodium phosphate buffer, pH 7.2. To wells of a 96-well plate with either 25 *μ*l of TTR or IAPP fibrils, 50 *μ*m newly dissolved TTR or IAPP in 0.05 M phosphate buffer containing 2% DMSO was added, followed by thioflavin T to 10 *μ* *M*. The final volume was 100 *μ*l. Fluorescence was measured every 20 minutes in a fluoroscan as described [28].

### 2.4. Statistics

Values are given as mean ± SD. Comparison between groups was performed with Mann Whitney test.

## 3. RESULTS

**Figure 1:**
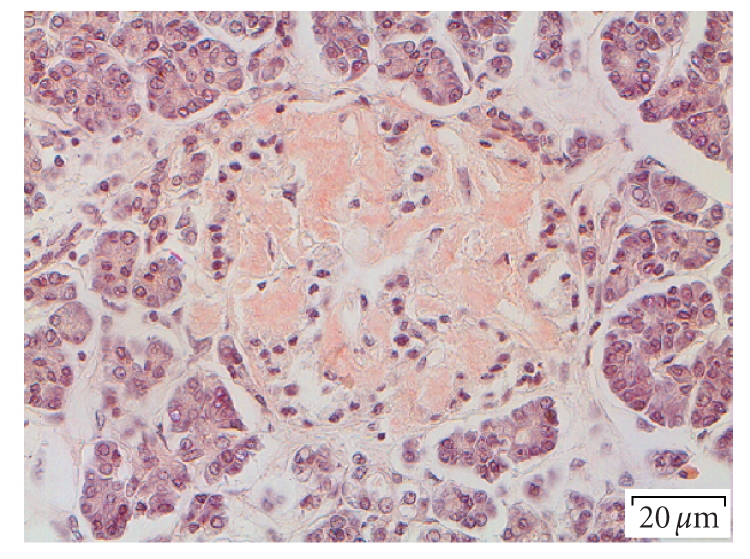
Islet in a type-2 diabetic patient. Most of the islet has been converted into amyloid; Congo red, bar 20 *μ*m.

In the pancreata of all individuals with type-2 diabetes and in 7 out of 10 individuals without diabetes, amyloid was found in islets of Langerhans. All of the diabetic individuals had a very pronounced islet amyloidosis with some islets with little remaining endocrine cells (Figure 1). In the nondiabetic patients, usually only small amyloid deposits were detected but in three individuals amyloid affected more than 20% and in one as much as 38% of the islets. Comparison of the percentage of affected islets in the two groups when only individuals with some degree of islet amyloidosis were included, showed that significantly more islets contained deposits in the diabetic individuals (98 ± 3% and 13 ± 14%, *P* = 0.001). The islet amyloid had typical staining properties with Congo red and was strongly labeled with antiserum against IAPP (not shown).

### 3.1. Hormone and transthyretin reactivity in islet cells of non-diabetic individuals

**Figure 2:**
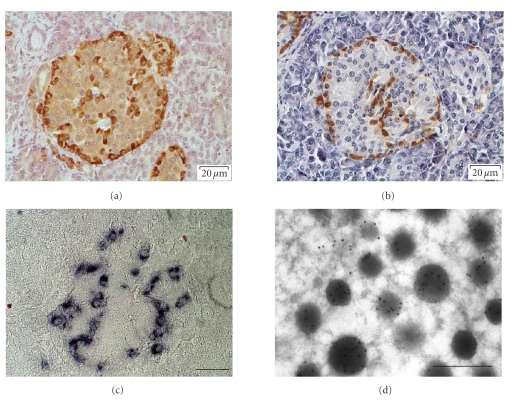
Normal human islets immunolabeled with antisera against: (a) transthyretin and (b) glucagon. In (c) is shown a normal human islet, subjected to in situ hybridization with a TTR probe, visualized with immunohistochemistry. Note that positive cells have a distribution indicative of glucagon cells. Bar 20 *μ*m. (d) shows a part of a glucagon cell with typical granules. The section was double immunolabeled for glucagon (10 nm gold particles) and TTR (5 nm gold particles). Immunolabeling for both these substances is seen on the granules, bar 500 nm.

In sections of pancreata without amyloid from nondiabetic individuals, both antisera A1898 and A1899 labeled islet cells in a similar way. A strong reaction was seen in all islets with cells with a preferentially peripheral distribution (Figure 2(a)) When compared with sections immunostained for glucagon, an identical distribution was seen (Figure 2(b)). In addition, the majority of remaining islet cells, mainly beta cells, were also labeled but only weakly (Figure 2(a)). In situ hybridization exhibited clear expression of TTR in cells which had a distribution of alpha cells only and no certain reactivity was found with beta cells (Figure 2(c)). Electron microscopically glucagon cells are characterized by secretory vesicles with a rounded electron dense core often situated somewhat eccentrically on the rest of the granule. Glucagon immunoreactivity occurred in both these areas of the granules while TTR labeling was mainly seen in the less electron dense parts of the alpha cell granules (Figure 2(d)). Small gold particles were also seen in the translucent areas of beta cells but only to a small extent (not shown.). Antisera against insulin and IAPP labeled normal beta cells as described [30].

**Figure 3:**
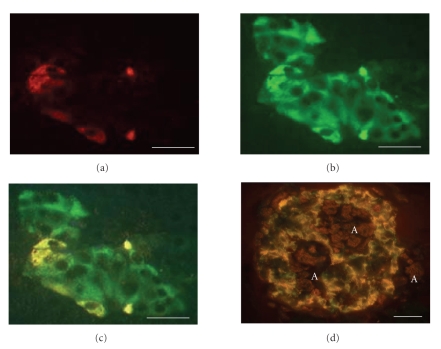
In (a), (b), and (c), an islet double labeled for glucagon (red) and TTR (green). Yellow color indicates colocalization. A large number of islet cells show TTR but not glucagon content. The islet in (d) is double labeled for insulin (green) and TTR (red). Many beta cells exhibit both TTR and insulin immunoreactivity (yellow). The amyloid (A) is weakly labeled for TTR. Bar (a)–(c) 25 *μ*m, (d) 200 *μ*m.

Double labeling for glucagon and TTR showed that all glucagon cells also exhibited TTR immunoreactivity (Figures 3(a) and 3(b)). In addition, a large number of islet cells, negative for glucagon, showed an evident TTR-reactivity (Figure 3(c)). Colocalization of insulin and TTR was seen in many, but not all, beta cells (Figure 3(d)).

### 3.2. Endocrine cells in islets with amyloid

Both insulin and glucagon immunoreactive cells were identified in islets will all degrees of amyloid deposits. As described earlier [31, 32], beta cells in amyloid-laden islets generally were devoid of IAPP-immunoreactivity. Many cells showed a strong labeling with antibodies against TTR. From adjacent sections, stained for TTR, insulin, and glucagon, it was obvious that not only alpha cells but also beta cells were strongly TTR-positive (Figure 3).

### 3.3. Transthyretin reactivity in islet amyloid

**Figure 5:**
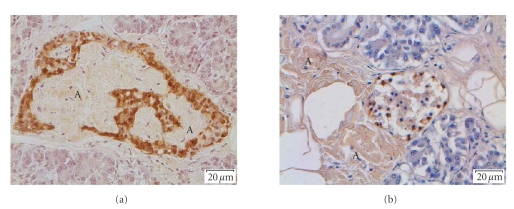
In (a) is shown an islet from a type-2 diabetic individual. There is pronounced IAPP amyloid infiltration, weakly labeled with antibodies against TTR. (b) shows a small islet in a nondiabetic individual with familial TTR-amyloidosis. There are heavy deposits of TTR-amyloid outside the islet but no islet amyloid. A = amyloid. Immunolabeled with antiserum against TTR.

Islet amyloid from diabetic and nondiabetic individuals exhibited a strong immunolabeling with antiserum against IAPP (not shown). Since we have shown previously that commercially available antibodies against TTR often do not recognize TTR in fibrillar (i.e., amyloid) form, we developed two different rabbit antisera which both strongly labeled cellular TTR and TTR in amyloid. The two TTR antisera labeled islet amyloid weakly in some cases (Figures 3(d) and 5(a)). This staining was even and no areas with strong reaction were seen. In the electron microscopic study, IAPP antiserum labeled amyloid in the diabetic case, but no certain binding of TTR antibodies to fibrils was seen. In order to study the specificity of the reaction in amyloid, we used an amyloid array with tissues from several patients in one block. The two TTR antisera showed reaction only with amyloid of known TTR origin (i.e., Swedish familial amyloidosis and SSA) but not with amyloid of AA, AL, AMed, or A*β* nature, showing that TTR-immunoreactivity is not a general feature of amyloid deposits.

The pancreatic tissue from the individuals with familial TTR-amyloidosis contained varying amounts of amyloid, irregularly distributed in the exocrine parenchyma and surrounding connective tissue. Although amyloid was seen very close to some pancreatic islets, TTR-amyloid did not occur within these islets (Figure 5(b)). Neither was IAPP-amyloid seen here.

### 3.4. IAPP fibrils seed fibril formation from TTR

**Figure 6:**
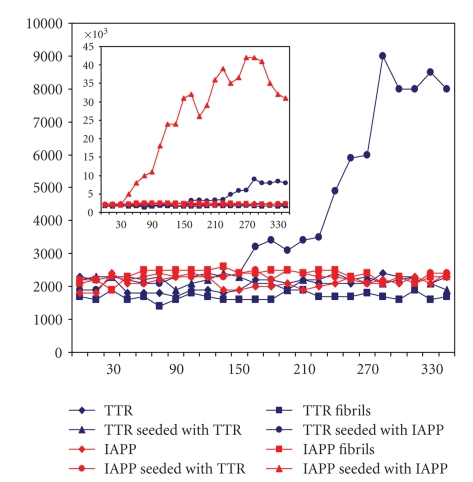
In vitro seeding and cross-seeding experiments with TTR and IAPP measured with thioflavin T. Preformed fibrils of IAPP seeded TTR (blue circles) and IAPP (red triangles, insert). No other increase in signals occurred. Thus, TTR fibrils did not seed IAPP (red circles).

Fibril formation of many amyloid proteins, including TTR and IAPP, occurs spontaneously in vitro after a lag phase which varies in length depending on protein. Seeding with preformed fibrils can shorten the lag phase considerably, which was seen most evidently when a solution of IAPP was seeded with IAPP fibrils (Figure 6, inserted). TTR fibrils did not induce any increase in fluorescence signal when incubated with newly dissolved TTR or solubilized IAPP (Figure 6). However, IAPP fibrils had a clear effect on TTR after a lag phase of about 3 hours (Figure 6).

## 4. DISCUSSION

This study confirms that TTR is normally expressed by pancreatic alpha (glucagon) cells. As earlier suggested [33], beta cells may also produce TTR but at a low degree, not detectable by in situ hybridization. TTR is stored in the secretory vesicles and is therefore most likely released together with hormones at exocytosis. TTR in plasma is a transporter of thyroxine and indirectly of retinol, but the function of the protein in the secretory granules is unknown. One can only speculate about the possibility that TTR binds to the glucagon precursor and may affect its processing.

IAPP and TTR are both well-known amyloid fibril proteins. Formation of amyloid fibrils is a nucleation dependent phenomenon which can be shown in vitro [14]. After a lag phase, which can last for several days, generation of fibrils usually is rapid. Seeding a fibrillogenic protein solution with preformed fibrils shortens or abolishes this lag phase. Cross-seeding, that is, the same effect achieved by addition of fibrils of a different biochemical nature, can occur with some proteins [16]. In this study, IAPP fibrils seeded IAPP efficiently. This was not seen with TTR which is in accordance with a previous report [34]. TTR fibrils did not seed IAPP but, interestingly, IAPP fibrils evidently induced fibril formation from TTR. Therefore, the weak immunoreaction of IAPP amyloid with antibodies against TTR in some cases may indicate existence of mixed fibrils in islet deposits as a result of a specific interaction between IAPP and TTR, although a passive diffusion of the latter molecule into the amyloid cannot be ruled out. While insulin is an inhibitor of IAPP fibril formation, effects of TTR is not known. It has been suggested that molecules may exist that lead to pathological protein folding and aggregation and these have been called “pathological chaperones” [35]. Therefore, the possible interaction between TTR and IAPP has to be studied further.

In the present study, we also evaluated the TTR immunoreactivity in alpha and beta cells in conjunction with type-2 diabetes. No such studies seem to have been published. Generally, islets in type-2 diabetic patients contained proportionally more strongly TTR-reactive cells in accordance with the known loss of beta cells in that disease [2, 3]. In addition, an interesting and unexpected finding was an increased TTR immunoreactivity of beta cells in pancreatic islets with heavy amyloid deposits. What this means is presently not understood but we have shown previously that IAPP in beta cells in islets with amyloid obtains altered immunoreactive properties [32]. Thus, IAPP immunoreactivity with a polyclonal IAPP antiserum was lost, although labeling with a monoclonal antibody was retained. This finding was interpreted as a sign of modification of the beta cell IAPP in type-2 diabetes. The finding here that IAPP fibrils interact with TTR is interesting in this respect. The observation in the present study that beta cells in amyloidotic islets are strongly TTR immunoreactive may indicate that the intragranular milieu is altered which may affect the ability of IAPP to aggregate.

## Figures and Tables

**Figure 4:**
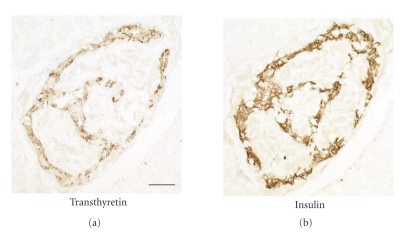
Amyloid-rich islet from a type-2 diabetic individual, in (a) immunolabeled for TTR, and in (b) for insulin. Note virtually identical distribution of immune reactive cells. No nuclear staining, bar 20 *μ*m.
